# An Exploratory Case Study of the Types of Resources Black Boys Use to Support Their Mental Health

**DOI:** 10.3390/healthcare10061082

**Published:** 2022-06-10

**Authors:** Brittany Ribeiro Brown, Ed-Dee G. Williams, Jamie M. Abelson, Arushi Chandrakapure, Daphne C. Watkins

**Affiliations:** 1 School of Social Work, University of Michigan, Ann Arbor, MI 48109, USA; eddeew@umich.edu (E.-D.G.W.); jabel@umich.edu (J.M.A.); daphnew@umich.edu (D.C.W.); 2 Department of Psychology, University of Michigan, Ann Arbor, MI 48109, USA; 3 Vivian A. and James L. Curtis Center for Health Equity Research and Training, University of Michigan, Ann Arbor, MI 48109, USA; arushic@umich.edu; 4 College of Literature, Science, and the Arts, University of Michigan, Ann Arbor, MI 48109, USA

**Keywords:** mental health, resources, social support

## Abstract

Black adolescent boys experience mental health challenges because of their exposure to a greater frequency and severity of psychosocial stressors. This study used a sample of Black boys at a high school in southeastern Michigan as a case study to understand the types of resources Black boys might use to support their mental health. After conducting a rigorous analysis of the study data using a rapid and an accelerated data reduction technique, four themes helped us answer the question: What kinds of mental health support resources are Black boys using? Four themes emerged from our analysis: *online resources*, *community and trusted individuals*, *self-reliance*, and *additional needs*. This case study is a springboard for further work to tailor a mental health education and support intervention, such as the YBMen Project, for Black boys and for building additional support amid the multiple crises occurring that impact their mental health and safety. Findings have implications for future research, practice, and policy to improve the mental health of Black boys in high school.

## 1. Introduction

Few studies examine the ways Black boys seek help for mental health challenges and their preferences for mental health support. The lack of research on mental health programming for Black boys is pertinent, given the increased likelihood that they will experience mental health challenges due to adverse life experiences such as racial discrimination, exposure to community violence, living in a low-income household, and attending underperforming schools [1]. Black male adolescents are impacted by multiple systems, including white supremacy, capitalism, and hegemonic masculinity. These interlocking systems impact Black boys’ mental health and their perceptions of mental health care. Though Black boys’ rates of clinical depression diagnosis are lower than that of other race and gender groups, Black boys often report more severe symptoms and prolonged experiences with depressive symptoms before receiving treatment and support [2,3]. One study found that 76.5% of Black youth have unmet mental health service needs, while other studies have estimated that rate to be 50% to 75% [4]. Overall, the mental health of Black boys is under-assessed, and they have significantly unaddressed mental health needs that require more attention [2,5].

Black boys, like other adolescents, are connected through multiple interacting social domains, which can promote positive development and mental health adjustment [6]. Though family, peers, school educators, and staff all have the potential to be systems of support for Black boys, they disproportionately face obstacles (e.g., access issues, insurance status, stigma, and barriers such as mistrust of the medical system) to obtaining mental health care when it is needed. For Black boys, addressing some of the contextual stressors (e.g., poverty, neighborhood violence, racial discrimination, etc.) that challenge their development provides a pathway to effective research and practice [7,8]. Additionally, adolescence is a time in human development when youth explore themselves in the context of their worlds and develop their understanding of who they are (i.e., their sense of identity).

Adolescence is a critical period when young people learn social and developmental skills for long-term psychosocial success. However, for Black adolescent boys, the freedom to fully experience the world and develop an understanding of their identity is disproportionately stunted due to their marginalized experiences, negative labeling, and lower expectations of success by society [9]. Despite their heightened risk of exposure to stressful life events, there is variability in Black boys’ mental health outcomes, providing evidence of their ability to navigate adverse situations and access resources successfully to achieve positive outcomes [7]. The social and emotional needs of Black boys are grossly understudied, calling for more research that explores how meeting the mental health needs of Black boys influences their mental health outcomes.

Despite the challenges experienced by Black boys in getting help for their mental health needs, few studies have examined the most relevant resources they use to achieve and maintain their mental health and well-being. Mental health resources are pivotal to developing positive self-identity for Black boys who confront widespread implicit bias and structural racism. Therefore, the purpose of this exploratory case study is to examine the mental health resources Black high school boys use to build additional resources that will support and sustain their mental health.

### 1.1. Mental Health Needs of Black Boys

The unaddressed mental health needs of Black boys are associated with unfavorable outcomes such as high rates of substance abuse, physical aggression, self-harm, and suicide. For more than a decade, mental health professionals have been concerned about the disproportionate increase in suicide among Black boys accounting for 80% of the suicides among Black youth [10]. Suicide ideation and attempts by Black boys increased by more than 200% between 1980 and 1995 and doubled from 1995 through 2012 [11]. Furthermore, data from the Centers for Disease Control and Prevention (CDC) reports that suicide rates for Black males are nearly four times the rate for Black females, with most of these suicides being committed by boys under the age of 18 [12,13]. More alarming, the rates are more likely to be misclassified (e.g., undetermined intent, suicide-by-cop) than whites [14].

Increases in suicide are related to several possible explanations, some supported by evidence and some that need more attention from researchers. Joe [15] speculated plausible reasons for a shift in suicide rates include: weakened community institutions such as the church; increased concentrated poverty which has impacts on environmental factors such as lead exposure; less traditional coping styles such as religious coping and external attribution orientation. Goodwill [16] found some support for the link between coping style and suicide. In a study with Black college students, self-blame/behavioral disengagement coping strategies were positively associated with suicide ideation, and religious coping strategies were negatively associated with suicide ideation [16]. Although there is a positive association between depressive systems and suicide ideation, Goodwill found that social support, self-blame, and religious coping strategies moderated the relationship [16]. Furthermore, Kurbin and colleagues [17] found support for the linkage between suicide with poverty and community intuitions in that deindustrialization of urban areas is associated with increased suicide rates of Black adolescents and emerging adults. They found that deindustrialization led to increased poverty and fewer opportunities for education, employment, and social mobility, impacting community, family, and social support that may protect against suicidality [17]. Moreover, anticipated, vicarious, and experienced racial discrimination has also been positively associated with depression and suicidal behavior [18]. This is alarming given that Black American youth experience between two to five vicarious events of racial discrimination per day, which has increased with access to the internet and social media [18,19,20].

While the rates of suicide among Black boys continue to grow, studies on Black boys’ mental health and help-seeking behaviors have provided mixed results. On the one hand, studies have suggested Black boys underutilize formal mental health support [21,22,23]. Yet, other studies have found that Black boys often seek support when experiencing high levels of mental distress, though it is typically from more informal resources such as family and peers [24,25,26]. The informal use of family and peers for mental health support helps Black boys overcome the stigma associated with mental illness and conditions such as depression [25,26]. Furthermore, studies have reported that high levels of social support from families, peers, and their community can act as a buffer between Black boys and severe mental distress and mental health stigma [26,27].

### 1.2. Families and Peers as Mental Health Support

The discourse around growing trends in Black boys’ mental health and suicide risk cannot exclude the importance of social connections Black boys experience during this critical developmental period. Social support affects health by regulating thoughts, feelings, and behaviors that promote health; fostering an individual’s sense of meaning in life; and facilitating health-promoting behaviors. As one of the most critical domains of human relationships, the capacity to establish and sustain relationships is a vital indicator of healthy psychological development. For example, friend support has been linked to improved physical and mental health, coping efficacy, and improved self-esteem [28]. Due to the reciprocity that occurs during social support, its positive components make it vital to mental health and well-being. Families and peers provide informal mental health support for boys who avoid formal mental health resources such as counselors and therapists [5]. Although Black boys prefer family and peers, studies by Lindsey and colleagues [21,29] found that Black boys with fewer familial ties or absent informal support structures often rely on formal mental health supports such as school counselors and clinical social workers, when in need.

Some scholars believe there are no connections between informal and formal support, particularly when it comes to Black boys. However, the two are connected through the *perspectives* of the people Black boys seek for help: their family and their peers [26]. When family members and peers share positive attitudes about formal support and encourage its use, Black boys are more likely to report favorable views and use it when experiencing mental distress [26]. Additionally, the role of the community is essential in supporting Black boys’ mental health needs. Breland-Noble, Burriss, and Poole [24] found that engaging with the communities surrounding and supporting Black adolescents is critical to promoting mental health treatment among Black adolescents. Specifically, engaging with the local community helped improve Black adolescents’ sense of trust and comfort with formal mental health support and increased participation [30]. Moreover, Heerde and Hemphill [31] conducted a meta-analysis on associations between informal help-seeking behavior, social support, and adolescent psychosocial outcomes and found similar results. They discovered that informal help-seeking was associated with decreased externalizing behavior and positively associated with other help-seeking behavior, and social support was associated with positive psychosocial outcomes [31].

### 1.3. Schools as a Place for Intervention and Support

When considering the mental health needs of Black boys, schools may be an ideal setting to implement effective mental health support programming. For instance, nearly 23% of youth with diagnosed mental health conditions received mental health treatment in the school setting compared to 20% that used outpatient services [32]. Similarly, other studies found schools to be uniquely suited to identify mental health challenges among students and support their mental health needs directly or through referral to external mental health services [33].

Mental health services are widely available in private and public schools and across racial/ethnic student populations; however, allocation disparities in access exist between rural and urban/metropolitan schools [34,35,36,37]. Schools are often in centralized geographic locations where young people spend most of their time, promoting access to connections and helpful resources. Therefore, proximity can also help to establish strong relationships with peers and school personnel such as teachers, principals, and counselors. Schools can provide a consistent and secure location for students experiencing pervasive mental health challenges [38,39], and they have the potential to expand their services for students with historically marginalized identities. The consistency in which youth are expected to attend school aids in assessing rapid changes in mood and behaviors that could indicate a mental health challenge. Support proximity can also establish strong relationships between students and school personnel such as teachers, principals, and counselors. Education and counseling psychology experts have argued that school-based mental health interventions not only help overcome physical barriers to mental health services (i.e., location and insurance status) but also ecological barriers (i.e., building relationships with the local community and involving parents, families, and peers in a student’s mental health support) [33,40,41,42,43,44]. Overcoming these barriers is essential to supporting the mental health needs of Black boys through tailored mental health resources. Though, the first step towards developing these tailored resources is learning about what Black boys are currently using for mental health resources.

### 1.4. Rationale for the Current Study

Although schools are considered an ideal location to support the mental health needs of youth, the matter becomes more complicated when thinking about school as an intervention site for Black boys. The primary challenge is the lack of previous research on the mental health of Black boys in school settings. In particular, few studies have examined Black boys’ use of mental health resources in schools. This is unfortunate since the school context can aid in adapting mental health services to better support the mental health needs of Black boys in the context of their preferred means for social support (i.e., family and peer support). Similarly, the importance of community and the effects of stigma on their help-seeking behaviors is another factor impacting how professionals can provide school-based mental health interventions for Black boys.

Some current literature has attempted to explain students’ unmet mental health needs, but it has fallen short when examining Black boys’ current preferences for mental health support. For example, recent research on mental health interventions for youth does not discuss Black boys and therefore overlooks their unique mental health needs and preferences [2,25]. This study aims to explore the mental health resources used by Black boys at a predominantly Black high school in southeastern Michigan. We used this exploratory case study to determine (1) what kinds of mental health support would be most helpful to Black boys and (2) the next steps for tailored and targeted programming specifically for Black male high school students.

## 2. Materials and Methods

### 2.1. Study Design

We used an exploratory case study design [45] for this study. We collected demographic and focus group data from Black male high school students to learn about their race and gender identities, their mental health needs, and the feasibility of developing a modified version of the *Young Black Men, Masculinities, and Mental Health* (YBMen) project [46,47,48] for Black high school boys in southeastern Michigan. The YBMen project [46,47,48] was launched in 2014. This psychoeducational, behavioral health intervention has been delivered as a social media-based mental health education and social support program for young adult Black men. It uses gender-specific, age-appropriate, and culturally sensitive prompts from popular culture (e.g., song lyrics, photos, YouTube videos, news headlines) to educate participants about mental health, healthy definitions of manhood, and social support [47]. This case study examines the mental health resources Black high school boys use in hopes of building additional resources that will support and sustain their mental health.

In addition to the focus group with students, we also held a focus group with school personnel—a small group of teachers, coaches, administrative staff, and social workers/school counselors—to obtain their input regarding the feasibility of implementing the YBMen intervention at their high school. The current study reports findings from the Black boys only, because the needs of this minority within a minority are rarely centralized in research efforts [46], and we sought to elevate their needs in this study. Future reports will include results from our analysis of both focus groups, allowing us to see what a tailored YBMen program might consist of for Black boys in high school in light of previous, successful YBMen programs among Black college men [48]. The institutional review board at the home university approved research protocols for the study.

### 2.2. Recruitment

We recruited, screened, and enrolled six Black male high school students to participate in this exploratory case study. Student homeroom advisory teachers first mentioned the focus group to their Black male students. If a student expressed interest in participating by informing the teacher, registering online, or telling a counselor, he was invited to meet with the TRAILS embedded counselor in a small group gathering, or one-to-one, to learn more. At that small group meeting, the counselor verified interest and eligibility, and questions were answered. At the end of the meeting, the young men received materials to take home, including parental consent and student assent form (two copies of each—one to return and one to keep) as well as a cover letter to explain this project and a two-page description of the YBMen program. The counselor contacted parents/guardians by phone to explain the project and answer questions. If interested, the parents completed the consent form and the students completed the assent form, returning both documents to the counselor at school. Students who participated in the focus group completed a brief demographic survey, verifying eligibility. Eligibility criteria were (1) a student at the identified high school; (2) an African American/Black male; and (3) willingness to discuss issues related to being a young Black man in a focus group setting.

### 2.3. Data Collection

During the fall semester of 2021, a newly graduated, Black male Ph.D.-trained researcher from our team facilitated the student focus group using the focus group questionnaire. An Indian-American female undergraduate research assistant helped. The focus group lasted one hour during lunchtime in a classroom at the high school. We served pizza. Only the focus group portion of the meeting was audio-recorded. Once the focus group was over, we gave the student participants $20 cash. One adult from the school sat in on the student focus group. The adult was someone the young men identified as someone they trusted. After the focus group, we sent a note to the participants and their parents to thank them for their participation and shared a link to a county-wide mental health resource list. Both the facilitator and the assistant took notes, and they completed a debriefing form after the focus group.

### 2.4. Data Analysis

The undergraduate research assistant transcribed the focus group recording and entered the demographic survey data into Microsoft Excel (Microsoft Corporation, Redmond, WA, USA). Qualitative data were analyzed using a team approach supervised by the lead investigator, who had extensive qualitative data analysis experience [49,50,51]. Coding allowed us to identify preliminary text segments to identify categories, concepts, and themes germane to the project goals. We analyzed the data using a rapid and an accelerated data reduction (RADaR) technique [50], and classical content analysis, which involved identifying the frequency of codes to determine which concepts were most cited throughout the data. Coding procedures allow segments of raw text to be identified and compared to other components. Meaningful text segments were used to identify categories, concepts, and themes relevant to the research questions and project aims.

## 3. Results

**Figure 1:**
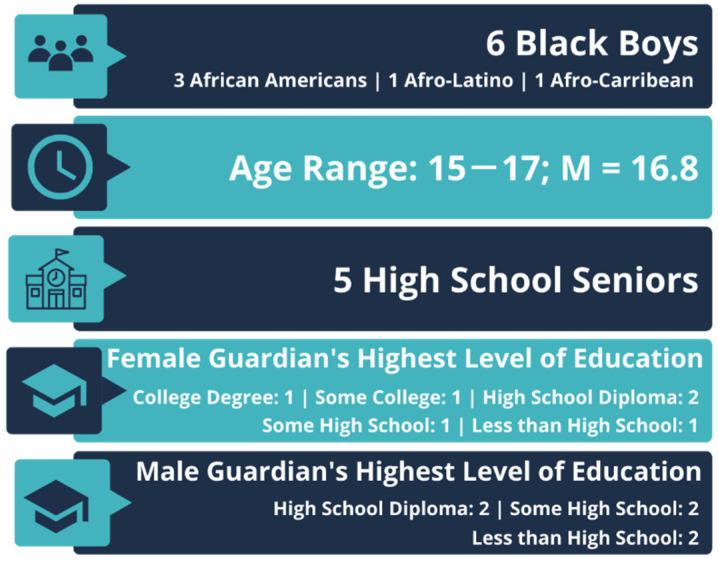
Sample Demographics.

This exploratory case study reports on the findings from a focus group we had with six Black adolescent boys in southeast Michigan (See Figure 1). The boys were all self-identified Black/African American, with one also identifying as Afro-Latino, and one also identifying as Afro-Caribbean. Although students from all grades were invited to participate, five of the boys who participated were in their senior year of high school, one was a sophomore, and their ages ranged from 15 to 17 years old, with a mean age of 16.5 years. Their parents’ or legal guardians’ highest level of education varied; female guardians had higher educational attainment than their male guardians. Female guardians’ education ranged from a college graduate, some college, high school diploma, some high school, and less than high school, with two with high school diplomas and one person in each of the other categories. Male guardians’ education had less disbursement: two participants’ parents had a high school diploma, two had some high school, and two had less than a high school education.

**Figure 2:**
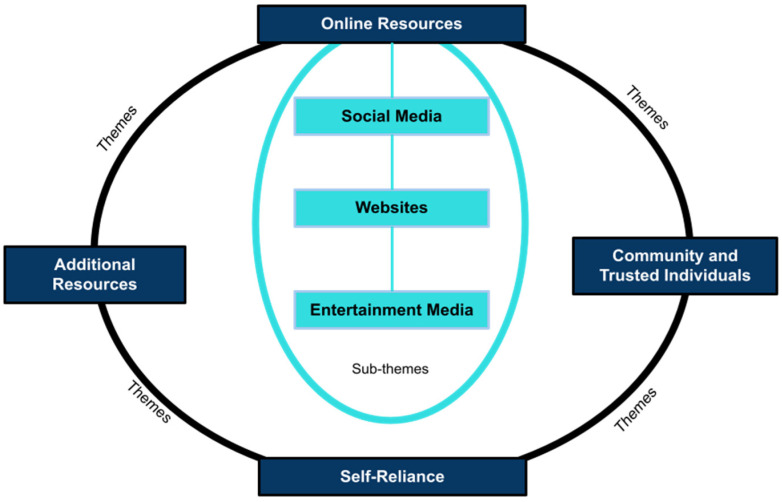
Study Themes and Sub-Themes.

The present study used an exploratory case study design [45]; thus, the focus group questions and analysis focused on learning more about where and how this specific group of Black boys used relevant resources to achieve and maintain their mental health. The analysis focused primarily on the following focus group questions: Where do you go for information? Who or what resources do you trust for health information? Where do you find information about stress? What kind of information do you all see about your health, like young Black men’s health? How do you keep up with what’s happening in the world today? What kind of social media do you all use? How do you keep up with what’s going on in your neighborhood? How do you feel about programs at your school? What characteristics would make a person or program a trusted source of information? Codes from our analysis were grouped into four central themes: *online resources*, *community and trusted individuals*, *self-reliance*, and *additional needs* (Figure 2). Online resources had three additional sub-themes: social media, websites, and entertainment media. Each is presented below.

### 3.1. Online Resources

The first central theme that emerged from our analysis was *online resources*. Given the broad nature of this theme, we categorized the codes that fell under this theme into three sub-themes: social media, websites, and entertainment media. The analysis identified media and websites as key sources of information on which the Black boys relied. “Media” included social media, celebrities, and entertainment, while “websites” referred to web pages such as Wikipedia and pages with a .edu or .org domains. When asked how they keep up with what’s happening in today’s world, their city, neighborhood, and school, one participant said: *“Social media, honestly, social media tells us everything.”*

#### 3.1.1. Social Media

Social media was disclosed by most of the boys as the go-to and primary source of information related to entertainment, their city, neighborhood, and school. They identified Instagram and Snapchat as their social media platforms of choice, and they described their usage as daily. There was no mention of Facebook or Twitter during the focus group. Though, not all participants primarily used social media as a source of information; two boys noted that they use social media less frequently. They made statements categorizing social media as a space for “drama.” The less frequent social media users described social media as *“Ain’t nothing entertaining.*”

Later during the focus group, when the boys were asked about the feasibility of a social media-based program like YBMen in their school for younger classmates, the boys responded positively that it would benefit younger boys like them. They said the use of social media is currently an issue at their school because peer pressure can sometimes have adverse effects:

*“... a lot of younger guys feel like they go on social media [and] they see the older guys [can] see what they doing…”*

However, they felt a program like YBMen could provide positive support to navigate negative peer pressure. One student noted:

*“Some people just tryna be just tryna fit in you guys got get the program sit them down and tell them be yoself you ain’t gotta prove nothing.”*

Two of the students agreed with this sentiment and followed up with:

*“They be like you know a lot of younger guys feel like they go on social media they see the older guys see what they doing they feel like it’s cool so they can do it that’s how people in real life get dying out here […]*

*“I agree with […] it’s like a lot of freshmen, sophomores, they tryna be something that they not but seeing stuff and just doing it”*

In response to these comments, one student noted the potential dangers of glamorizing people’s struggles on social media:

*“..they tryna glamorize it it’s like it’s not something to glamorize. Some people actually go through that type of stuff.”*

#### 3.1.2. Websites

The second sub-theme under online resources was websites. When we asked who or what sources the Black boys trusted for health information, participants noted that websites with .org or .edu domains were trustworthy domains, and websites like Wikipedia were untrustworthy.

*“I only do .org, .com and everything like that. I don’t get nothing of Wikipedia nothing like that.”*

Another student voiced his perspective:

*“Cuz mostly it’s just informational not really confrontational, so it’s more about actual facts and studies less [like] stuff [they] promote [so you can] get an item that you probably don’t want or something that people say works for them, but it might not work for you.”*

This response alludes to the mass amount of information and less trustworthy information on the web. The students felt that one must sift through and assess these resources for accuracy and trustworthiness. Another student had similar sentiments:

*“Everything on the internet [is] false information...”*

#### 3.1.3. Entertainment Media

The final sub-theme under online resources was entertainment media. In addition to social media and websites, participants named entertainment media and celebrities as harmful sources of information.

*“A lot of people see celebrities as role models. That’s they that’s they purpose. Influencers.”*

When asked about what they would like to see in a program like YBMen at their school, one student said:

*“I think y’all should talk about the things that really hurting our community that no one wanna talk about, especially on the media.”*

Similarly, another participant raised concerns about role models and how they do not reach Black boys at their school:

*“The role models they put in front of us. That we see every time we open our phones or turn on the TV. It’s a lot; it’s a lot. The way the system is, the way they teach us the things they teach us is just all messed up.”*

Discussions about the role of entertainment media also occurred in the context of what needs to happen for Black boys to truly have what they need to succeed in life. Models are essential, and variations in these models are ideal.

### 3.2. Community and Trusted Individuals

The second central theme that emerged from our analysis was community and trusted individuals. Some participants expressed relying on community and trusted individuals for support. One student who said he was less likely to use social media to keep up with information about his community said he would talk to his neighbors to get the information he needed, or simply “be outside.” He said:

*“I just, I have a connection with the people around me in my neighborhood, so I just … talk … to them outside….. Get they opinion on it, how they feel about what’s going on...[…]...that’s where I get my info from.”*

The participants also discussed community spaces as a place where one would want to receive health information. When asked where and how a program like YBMen should be implemented, one student responded: *“Honestly, I would say probably like this but maybe on a bigger scale in the library or the auditorium. So set meetings to have after school.”* Another student followed up with a similar sentiment and said: *“I would say fairs. Like fairs and everything. Cuz it’s more likely that they gonna come to a fair than just a meeting.”*

Participants also emphasized the characteristics of trusted individuals. Specifically, when referring to the infrastructure of a program like YBMen, they indicated that a trusted leader should run it. They defined this person as someone with shared lived experiences: *“I think it [the program] would benefit if it comes from somebody that grew up the same way we did and they at a good place in life now...”* To further illuminate this point, another participant said,

*“Obviously, [the person will] have to be educated but yeah kinda goes back to what they were saying like kinda grew up the same way and they’re at a stable point now like possibly role models from our community that’s also educated cuz you don’t just want no random dude coming up you know yeah….”*

One of the participants went as far as to identify a former staff member as an exemplary person to run a program and said,

*“…cuz when I would talk to him he would always like to throw some like life examples like yeah when I was little or stuff like that like he would say something he did how he acted what he just like what he did cuz he like really he was he was struggling and I and I look at how he is now how he successful he like he was the dean.”*

Some of the boys explained, more specifically, who were trusted individuals in their own lives, which included family members and older individuals. One participant shared, *“I just … talk to people that’s older than me that know more than me.”* While another participant said,

*“Any stress-related stuff like I just talk to older people that’s like been through a lot to get they knowledge. Like everyone has different experiences with dealing with stress and ain’t always gonna be the same for everybody.”*

In addition, a different participant spoke about the men in his family, and how he reaches out to them when he needs support:

*“If I get stressed out, I probably talk to my Dad or talk to my brothers. Cuz they’re wise people, so I can have really deep conversations with them and get my mind off of stuff.”*

### 3.3. Self-Reliance

The third central theme that emerged from our analysis was self-reliance. Some boys disclosed a distrust of others and a reliance on the self for psychosocial support. When asked about utilizing community and neighborhood engagement for information, one student said, *“I stay to myself.”* Later in the focus group, when the facilitator probed why the boys felt some websites were trustworthy, another student responded, *“I trust my own gut.”*

When we asked the focus group’s participants to share where they go to learn about stress, participants responded with a variety of different responses, ranging from not feeling stressed (i.e., *“I just be chillin. I don’t really get, I don’t trust no one…”*) to the importance of knowing how to manage stress on your own.

One participant said, *“You know dealin with stress is like you know that’s 101. That between you and you, you feel me? There’s nobody else that can really help you.”*

Immediately after, another participant shared, *“I agree … yeah, … it depends on how you feel cuz it really is your stress, you can’t put it on the other people.”*

Another student alluded to a culture of self-reliance from many Black boys at their school. In response to why students might not show up to a program, one student said:

*“… the subject … you don’t wanna talk about your problems cuz it’s real sensitive to you and depends on what you go through … so you might not wanna bring stuff back up that happened to you so even though you may need [the YBMen program] … it’s probably … something you don’t want to put on other people … you just do it cuz like you afraid of feeling that type of pain again.”*

### 3.4. Additional Needs

The final theme that emerged from our analysis was additional needs, including other topics our participants discussed as important to their mental health. Specifically, participants identified psychosocial needs throughout the focus group that are not being met due to a lack of culturally tailored programs, messaging, education, and care. When asked what kind of information they see about Black males’ health, one student responded: *“Honestly, I don’t see nothing...”* Another focus group participant expressed needing role models and more leadership in Black communities, generally, people Black youth can look to for guidance. He shared,

*“... we need more leaders and if we could [get them]… that would be great … cuz it’s like everybody needs a role model … it would be nice to have someone to look up to.”*

During the discussion about additional needs, students also shared that they wanted more support offered by their school. In particular, the students expressed concern that their school was not preparing them for adulthood and at times caused harm.

*“I feel like the main thing, in my opinion, is that they don’t really have classes or things … that are set up for becoming adults or going into adulthood. It’s like yeah you learn some stuff naturally, but like a lot of people struggle outta high school. “*

Other students had adverse reactions to the role of the schools in the development of Black boys, and they raised concerns about the school-to-prison pipeline (i.e., *“They training us to go to prison.”*). The boys also acknowledged that the judicial system was where many of their friends and classmates ended up after high school (i.e., *“Cuz most people after they leave they either end up dead or they go to jail.”*). A poignant need that the students expressed was wanting more programs specifically tailored for Black boys (i.e., *“We need more programs that show young Black men how to live instead of how to survive.”*)

## 4. Discussion

This paper aimed to report the findings from an exploratory case study of Black boys at a southeastern Michigan high school. We learned how Black boys are impacted by capitalism, patriarchy, and white supremacy. These oppressive systems were reflected by the issues they faced with respect to lack of positive role models, stress, lack of perceived support from school, and community violence. We wanted to learn where and how Black boys use relevant resources to achieve and maintain their mental health. We learned a lot about their needs and who—and what—they turn to for support. Our analysis uncovered four themes (online resources, community and trusted individuals, self-reliance, and additional resources) that demonstrate where Black boys turn for mental health resources.

It is no surprise that Black boys turn to online resources when seeking mental health support. In our study, participants reported using social media, websites, and entertainment media to educate themselves about mental health concepts and to decide where to go for help. Participants’ responses reflect a wide range of how Black boys relate to and use social media. Our participants mentioned using Snapchat and Instagram [52] and not Facebook or Twitter. These comments reflect a national trend, suggesting that Snapchat and Instagram are the most used platforms by people under the age of twenty-four [53]. A primary takeaway from this finding is that social media-based interventions may need to pivot platforms based on the age group of their service users. Naturally, as social media preferences change, practitioners must update delivery methods.

Our findings regarding the contradiction between Black boys reporting that they prefer online resources yet do not trust online resources corresponds with results from a systematic literature review that found adolescents generally mistrust health information online but still use it [54]. Additionally, research has found that adolescents are less trustful of social media health information than health information found on websites [54]. These corroborating findings suggest psychoeducation and mental health intervention efforts should strive to equip adolescents with online health literacy skills to have the tools necessary to seek, discern, and find trustworthy online information.

Black boys from our study noted the critical role of entertainment media in the lives of our participants. Responses reflected a desire for Black male role models to talk about issues related to Black male wellness. This was seen in previous literature, particularly from previous YBMen programs [49]. There are positive psychosocial outcomes when Black males have a male role model [55]. Our respondents identified current role models (e.g., celebrities or influencers) who do not fulfill this need and who often perpetuate harm by not setting good examples for young Black, impressionable boys. Additionally, these responses concerning entertainment media indicate that they have media literacy skills (knowledge of media production, framing, and influence), and one study found that media literacy among Black viewers is associated with increased self-esteem [56]. So, while it may be challenging to change trends in entertainment media, health practitioners should incorporate opportunities to interpret media (i.e., health media literacy) into programming for Black boys.

For our study, community and trusted individuals included neighbors, family members, friends, and school staff. Black boys from our study reported seeking out trusted individuals whenever they needed mental health support. These findings are aligned with previous studies suggesting that Black boys seek help for their mental health challenges [24,25,26]. Trusted individuals are often role models and other trusted people who guide adolescents’ beliefs, values, and behavior [55]. In our study, trusted people included people with a certain level of relatability who the boys perceived as having similar experiences but prevailed through life’s obstacles. These findings suggest that future programs for Black adolescents should include perceived successful leaders from the community or similar communities.

Self-reliance is considered a positive coping mechanism for Black adolescents and men [57,58]. However, it may also be an impetus for seeking support, and it is associated with adverse health outcomes [59,60]. In the current study, self-reliance referred to the distrust of others and it expressed the need to rely on oneself. In a study that examined coping strategies of Black and white adolescents, self-reliance was among the top three coping strategies of the twelve measured, and Black adolescents used this strategy more than their white counterparts [61].

Our final theme (i.e., additional needs) reflects Black boys’ mention of resources they do not have access to or desire. In the city where our high school students reside, Black students are 1.9 times as likely to be suspended as White students [62]. As students, Black boys are more likely than boys of any other race or ethnicity to be suspended, expelled, punished, referred to law enforcement, or arrested for in-school behavior. Structural racism and discrimination are overlooked factors that play a prominent role in the overrepresentation of Black boys in systems, particularly when mental health challenges are involved [58]. Yet, due to historical biases and social injustices that disproportionately affect the lives of Black boys, society frequently blames them for the challenges they experience navigating life transitions and trajectories. Improving existing systems to benefit Black boys requires a multifaceted approach that must include strategies to enhance access to effective, culturally-affirming social and emotional support for Black boys.

School professionals often perceive Black boys’ expressions of stress, anxiety, and depression as irredeemable behavior. The resulting school exclusionary discipline practices, such as suspensions, expulsions, and office disciplinary referrals, alienate and segregate Black boys from the school and the community, creating barriers to learning and academic achievement. The practices also lead to involvement in the judicial system, poor mental health, and harmful outcomes over the life course [63]. However, there is promise and potential for school-based interventions for Black boys. A study by Bains, Franzen, and White-Frese [43] reported that Black boys preferred school-based mental health resources over community-based mental health resources when experiencing mental health challenges. The authors identified convenient access to support; the individualized support they received, having established relationships with school staff; and feeling secure in the school as key factors that made the boys not only prefer school-based resources but also be more receptive to help, overcoming the stigma that prevents them from seeking mental health support in the first place.

### Study Limitations

Despite the insight we garnered from this exploratory case study, the findings should be interpreted in light of a few limitations. First, we collected the data during the COVID-19 pandemic, and the school was under unusual stress and pressure. Second, given the nature of this exploratory study, only a small number of Black boys from one high school in southeastern Michigan were included. Though we were intentional with our decision to sample a small number of students for this exploratory case study, we recognize that a more robust qualitative sample would garner more attention from specific academic and research communities. This study was the first step to achieving our overall goal: to tailor and target a mental health education and social support program developed for Black college men (i.e., the YBMen project) for Black male high school students. Thus, the findings from this exploratory case study will inform more comprehensive, qualitative, and quantitative research on Black high school boys and the future of mental health programming that can positively impact their lives.

Third, we purposely did not include the data from the focus group with teachers and school personnel in this study. We wanted this study to centralize Black male students’ experiences, needs, and voices. Early studies show that teachers do not often perceive their Black male students positively. Their perceptions of Black boys are often through racial stereotypes that disproportionately refer Black boys for disciplinary action [64,65]. Despite this, however, we recognize that teachers and other school personnel play valuable roles in the lives of Black male students. The focus group data from the teachers, coaches, counselors, and school administrators will be included in future analyses. These data will help us inform future programming for Black boys and their high school.

Finally, our sample consisted of five high school seniors and one sophomore. As a result, we may be missing perspectives from high school boys from various grades who may have different salient needs than students in their last year of school. For example, themes of *self-reliance* and *community & trusted individuals* could look different for adolescents in their first year of high school. The boys may have a different relationship with seeking social support, and they may have not yet learned the consequences of trusting the wrong people. In fact, during the focus group, the seniors spoke of the younger guys as not having known who were viable role models. The seniors in the focus group, perhaps starting to think about life after high school, shared a desire to learn more about how to better function in life outside of school: *“They would rather give you 400 pages of reading than teach you how to tie your shoes.”*

## 5. Conclusions

There is a need for a deeper understanding of the social and emotional needs of Black boys, such as experiencing meaningful relationships and their influence on mental health adjustment. Researchers and practitioners are primed and ready to suggest programming that can provide a buffer of protection for Black boys that have experienced toxic stress due to ongoing physical and psychological trauma. However, sustainable and scalable programs build on preliminary work to generate science informing program measures and outcomes. This study sought to take the first step in developing science that would inform the adaptation of the YBMen program with Black high school boys. Strong programming efforts toward Black boys will deepen our understanding of the social connections that genuinely protect their ability to cope with stress.

## Data Availability

Data supporting reported results can be found at the University of Michigan School of Social Work Vivian A. and James L. Curtis Center for Health Equity Research and Training.
